# New retinal findings in *NLRP3*-associated autoinflammatory disease

**DOI:** 10.1186/s13023-023-02815-1

**Published:** 2023-07-21

**Authors:** Zhangwanyu Wei, Zhikun Yang, Donghui Li, Xiao Zhang, Bing Li, Xufeng Zhao, Wenyu Yan, Bingxuan Wu, Na Wu, Xuqian Wang, Weihong Yu, Min Shen

**Affiliations:** 1 Present Address: Department of Ophthalmology, Peking Union Medical College Hospital, Beijing, 100730 China; 2 Key Laboratory of Ocular Fundus Diseases, Chinese Academy of Medical Sciences, Beijing, 100730 China; 3 Department of Rheumatology and Clinical Immunology, National Clinical Research Center for Dermatologic and Immunologic Diseases (NCRC-DID), Ministry of Science & Technology, State Key Laboratory of Complex Severe and Rare Diseases, Peking Union Medical College Hospital, Key Laboratory of Rheumatology and Clinical Immunology, Chinese Academy of Medical Sciences & Peking Union Medical College, Ministry of Education, Beijing, China

**Keywords:** *NLRP3*-associated autoinflammatory disease (*NLRP3*-AID), Cryopyrin-associated periodic syndrome (CAPS), Retina, Retinal vascular leakage, Macular ischemia, Microaneurysms

## Abstract

**Purpose:**

To determine whether the rare NLRP3-Associated Autoinflammatory Disease (*NLRP3*-AID) is associated with retinal changes and to assess the ocular involvement.

**Methods:**

A retrospective cohort study of 20 patients(40 eyes) diagnosed with rare NLRP3-AID at Peking Union Medical College Hospital, from April 2015 to August 2022. Patients underwent a comprehensive ophthalmological examination, including visual acuity, intraocular pressure examination, slit-lamp examination, fundus photography, optical coherence tomography(OCT), and fluorescence angiography (FA). Some patients also underwent optical coherence tomography angiography (OCTA).

**Results:**

This study analyzed 40 eyes of 20 patients (11 [55.0%] male; median age, 25.0 years [range, 12–52 years]) and 13 patients (26 eyes, 65%) demonstrated ocular involvement. The most common ophthalmologic manifestation was conjunctivitis (22 eyes, 84.6%), followed by papilledema (14 eyes, 53.8%), retinopathy (10 eyes, 38.5%), optic atrophy (6 eyes, 23.1%), uveitis (4 eyes, 15.4%), reduced pupil light reflex (3 eyes, 11.5%) and cataracts (2 eyes, 7.7%). Ocular involvement was bilateral in 11 patients (55.0%). Five kinds of retinal lesions were seen in 5 patients (10 eyes, 25%) with *NLRP3*-AID, including peripheral retinal vascular leakage, microaneurysms, macular ischemia, macular epiretinal membrane formation and drusen.

**Conclusions:**

Peripheral retinal vascular leakage, macular ischemia, microaneurysms and drusen are newly identified retinal findings in patients with *NLRP3*-AID, which suggests the importance of detailed retinal examination in these patients.

## Introduction

*NLRP3*-associated autoinflammatory disease (*NLRP3*-AID), often referred to as cryopyrin-associated periodic syndrome (CAPS), is a rare, heterogeneous disease entity caused by variants in the NLR family pyrin domain containing-3 (*NLRP3*) gene on chromosome 1q44 [1]. *NLRP3*-AID, which affects one to three in a million children and adults worldwide,[1, 2] is characterized by repeated episodes of fever and a variety of inflammatory signs [3, 4]. The longstanding uncontrolled inflammation results in irreversible organ damage, including sensorineural hearing loss, amyloidosis, vision loss, skeletal deformities and cognitive disability [1].

Previous publications have noted that 71% of patients with *NLRP3*-AID demonstrate ophthalmologic manifestations [5, 6]. Patients with *NLRP3*-AID have been reported to present with conjunctivitis, uveitis, papilledema, optic atrophy, cataract and glaucoma [5, 7]. To the best of our knowledge, retinopathy in *NLRP3*-AID patients has rarely been documented. In our previous study, we identified a middle-aged woman with *NLRP3*-AID and early onset drusen [8]. Retinopathy in *NLRP3*-AID is a surprising finding that deserves attention, as it could help deepen the understanding of this disorder. To date, we have found five kinds of retinal changes in *NLRP3*-AID patients.

The objectives of the present study were to report four newly recognized kinds of retinopathy in *NLRP3*-AID patients, as well as to provide all the ophthalmologic manifestations of *NLRP3*-AID in a single-center cohort of *NLRP3*-AID patients diagnosed after 2015.

## Methods

### Study participants

All participants provided written informed consent. The study was approved by the Peking Union Medical College Hospital ethics committee (JS-3421) and performed according to the Declaration of Helsinki. A total of 20 patients were diagnosed with *NLRP3*-AID at the Department of Rheumatology and Immunology, Peking Union Medical College Hospital, from April 2015 to August 2022. The diagnostic criteria for *NLRP3*-AID included (1) elevated inflammatory markers (CRP/SAA) and (2) at least two of six typical symptoms (urticaria-like rash, cold-triggered episodes, sensorineural hearing loss, musculoskeletal symptoms, chronic aseptic meningitis and skeletal abnormalities) [1]. All the participants met the above diagnostic criteria and underwent genetic tests.

### Ophthalmic evaluations

All participants underwent comprehensive ophthalmic evaluations, including best-corrected visual acuity testing (BCVA), intraocular pressure (IOP), slit-lamp examination, ophthalmoscope examination, fundus photography, fluorescein angiography (FA) and optic coherence tomography (OCT). Color fundus images were obtained using the Visucam 224 (Carl Zeiss Meditec, Dublin, CA), FA was performed using the Spectralis HRA + OCT device (Heidelberg Engineering, Heidelberg, Germany), and OCT examinations were performed using a VG200 SS-OCT (SVision Imaging, Ltd., Luoyang, China). Some patients also underwent optical coherence tomography angiography (OCTA) examinations (VG200; SVision Imaging, Ltd., Luoyang, China) and visual field testing (Humphrey Field Analyzer, Zeiss, Dublin, CA).

### Statistical analysis

The incidence of ocular manifestations was calculated form the total number eyes of participants (n1 = 40). The frequency of each manifestation was calculated from the total number of eyes with manifestations (n = 26).

## Results

### Ocular manifestations in patients with *NLRP3*-AID

This study analyzed 20 patients (11 [55.0%] male; median age, 25.0 years [range, 12–52 years]). Ocular involvement was observed in a total of 13 patients (26 eyes, 65%). Twelve of them had confirmed variants in the *NLRP3* gene. One patient was *NLRP3*-variant negative but had characteristic clinical features of *NLRP3*-AID. The most common ophthalmologic manifestation was conjunctivitis (22 eyes, 84.6%), followed by papilledema (14 eyes, 53.8% ), retinopathy (10 eyes,38.5%), optic atrophy (6 eyes, 23.1%), uveitis (4 eyes, 15.4%), reduced pupil light reflex (3 eyes,11.5%) and cataracts (2 eyes, 7.7%). Eleven patients manifested bilateral ocular involvement (55.0%).

### Retinal changes in patients with *NLRP3*-AID

Five patients showed distinct retinal changes. Among them, two patients manifested peripheral retinal vascular leakage, while scattered microaneurysms, macular ischemia, focal epiretinal membrane, and macular drusen were found in one patient each (Table 1).

**Table 1:** Demographics and clinical characteristics of *NLRP3*-AID patients with retinal findings

Patient No.	1	2	3	4	5
Gender	F	M	F	M	F
Age at onset, years old	2	6	3	2	7
Age at diagnosis, years old	39	32	12	20	18
BCVA (OD)	20/25	20/20	20/200	20/20	20/20
BCVA (OS)	20/25	20/20	20/32	20/20	20/20
Family history	+	+	+	-	-
Conjunctivitis	+	+	+	-	+
Uveitis					
Papilledema	+	+	-	+	-
Optic atrophy	-	-	+	-	-
Retinopathy	+	+	+	+	+
*NLRP3* variants	p.T348M	p.G326E	p.A439V	p.K829T	-

*NLRP3*-AID, *NLRP3*-Associated Autoinflammatory Disease; No., number; BCVA, best corrected visual acuity

Patient 1 was a 39-year-old Chinese woman who had *NLRP3*-AID with widespread drusen at the posterior poles of both eyes; she was described in our previous case report [8].

**Fig. 1:**
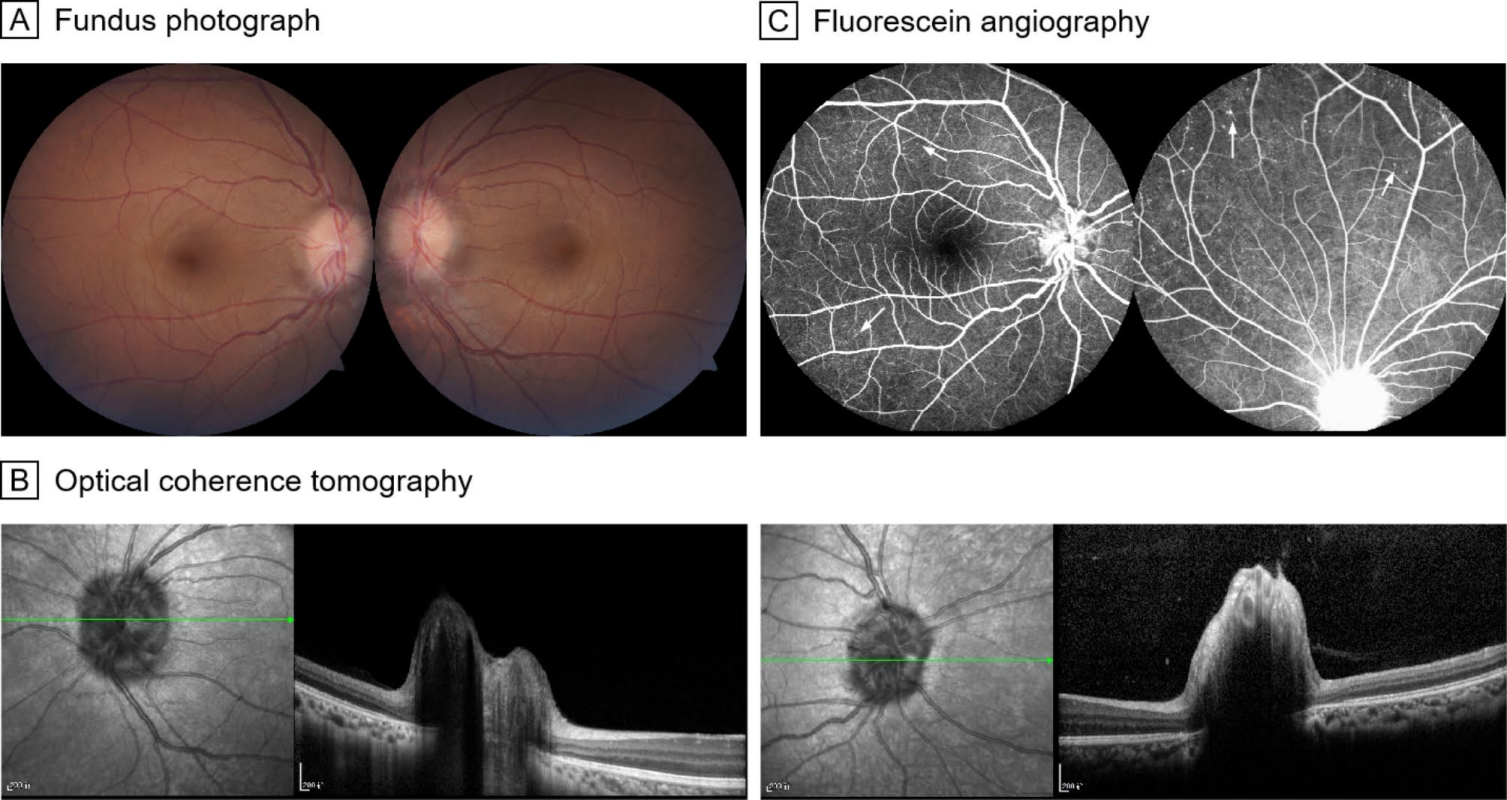
Fundus photograph, FA and OCT of patient 2. **A** and **B**, Fundus photographs and OCT reveal papilledema in both eyes. **C**, FA images demonstrate early-stage hyperfluorescence of the optic disc in both eyes and scattered microaneurysms (white arrows) in the mid-peripheral retina of both eyes

Patient 2 was a 32-year-old Chinese man who presented with recurrent rash and fever since the age of six. He was first admitted to the Department of Rheumatology and then referred to the Department of Ophthalmology due to red eyes. Best corrected visual acuity (BCVA) was 20/20 bilaterally. Ophthalmoscopy and OCT revealed papilledema in both eyes (**Fig. **1AB). FA examination demonstrated early-stage hyperfluorescence of the optic disc in both eyes and scattered microaneurysms in the mid-peripheral retina of both eyes (Fig. 1C).

**Fig. 2:**
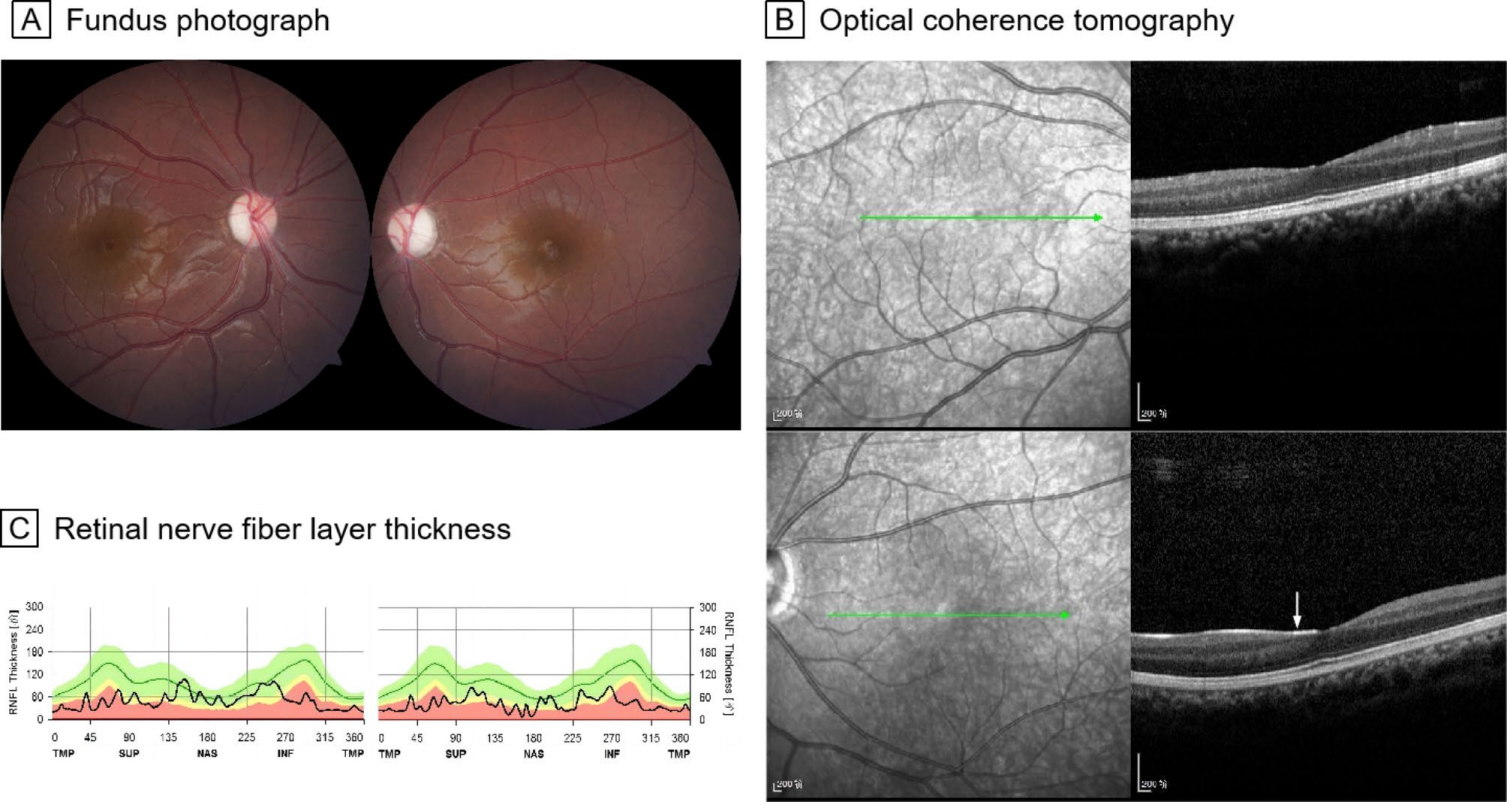
Fundus photograph, OCT and retinal nerve fiber layer thickness of patient 3. **A**, Fundus photographs show pale discs in both eyes. **B**, OCT images shows a focal epiretinal membrane (white arrow) in the left eye. **C**, Retinal nerve fiber layer thinning was found in both eyes

Patient 3 was a 12-year-old Chinese girl who presented with recurrent rash and fever since the age of three. BCVA was 20/200 in the right eye and 20/32 in the left eye. Ophthalmoscopy examination showed pale discs in both eyes (Fig. 2A). A focal epiretinal membrane in the left eye was also found on OCT (Fig. 2B). OCT demonstrated thinning of the retinal nerve fiber layer in both eyes, which was consistent with optic disc atrophy (Fig. 2C).

**Fig. 3:**
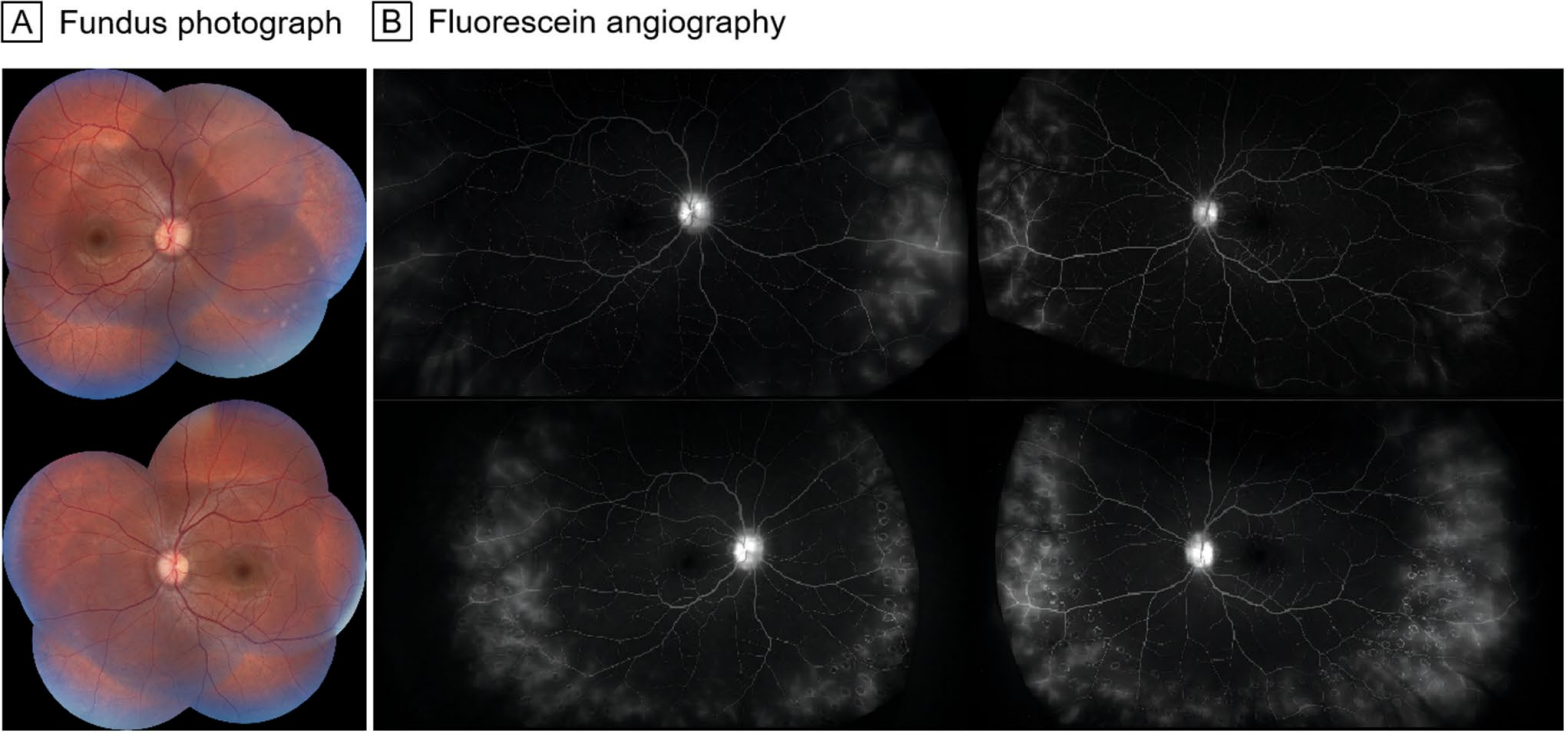
Fundus photograph and FA of patient 4. **A**, Fundus photographs show mild papilledema without obvious retinal changes. **B**, FA (top row) reveal vascular leakage at the peripheral retina of both eyes. Two months after retinal laser photocoagulation (bottom row), there is no improvement in vascular leakage

Patient 4 was a 20-year-old Chinese man who presented with recurrent rash and fever since the age of two. BCVA was 20/20 bilaterally. Ophthalmoscopy showed mild papilledema without obvious retinal changes (Fig. 3A). FA revealed vascular leakage at the peripheral retina of both eyes (Fig. 3B). Scattered laser treatment at the peripheral retina vascular leakage area and sub-Tenon’s capsule injection of 20 mg triamcinolone acetonide were performed bilaterally to attempt to alleviate the leakage. However, two months after retinal laser photocoagulation, when we repeated the FA, there was no improvement in the vascular leakage. He then received 150 mg of canakinumab subcutaneously every 8 weeks. Although his clinical symptoms improved remarkably after the treatment, unfortunately, the vascular leakage had not resolved according to FA.

**Fig. 4:**
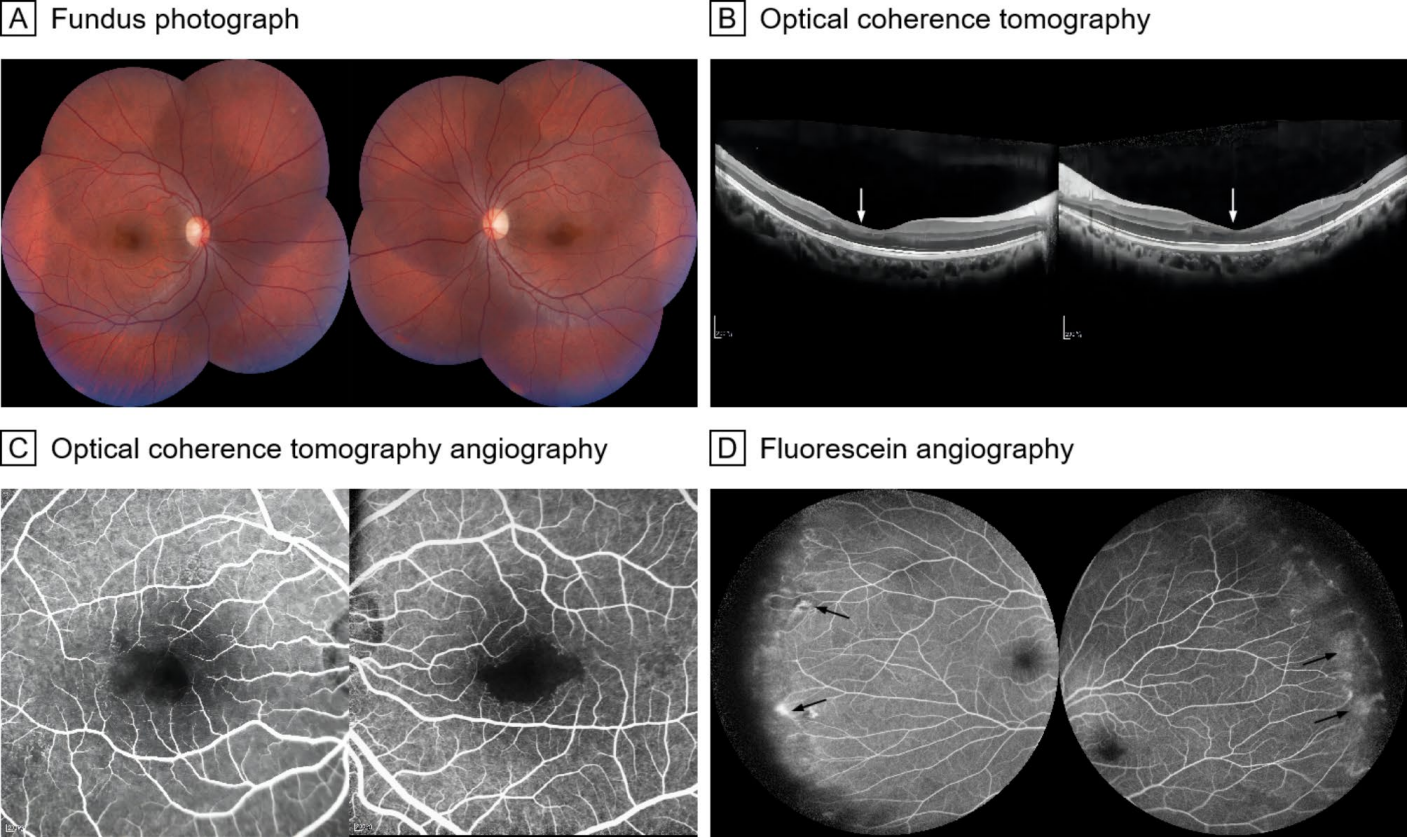
Fundus photograph, OCT, OCTA and FA of patient 5. **A**, Fundus photographs show no obvious retina changes. **B**, OCT images demonstrate disruption of the outer plexiform layer (white arrows). **C**, OCTA images demonstrate an enlarged and irregular macula foveal avascular zone and nonperfusion area. **D**, FA showed vascular leakage (black arrows) in the periphery of the retina

Patient 5 was an 18-year-old Chinese woman with a history of blurred vision for two years. The BCVA was 20/20 bilaterally. Ophthalmoscopy showed no obvious retina changes (Fig. 4A). OCT demonstrated disruption of the outer plexiform layer (Fig. 4B). OCTA demonstrated an enlarged and irregular macula foveal avascular zone and nonperfusion area (Fig. 4C). FA showed vascular leakage in the periphery of the retina (Fig. 4D).

The retinal findings of *NLRP3*-AID in our study were variable, both clinically and genetically. Patient 1, who presented with drusen, carried pathogenic *NLRP3* (NM_001243133.1) variant T348M. Patient 2, who presented with microaneurysms, carried pathogenic variant G326E. Patient 3, who had an epiretinal membrane, carried pathogenic variant A439V. Patient 4 showed peripheral retinal vascular leakage on fluorescein fundus angiography (FA) and carried pathogenic variant K829T. Patient 5 demonstrated peripheral retinal vascular leakage, nonperfusion and macular ischemia; despite her clinical diagnosis, however, she carried no known *NLRP3* variant.

## Discussion

To the best of our knowledge, this is the first study reporting four new retinal findings in *NLRP3*-AID, including peripheral retinal vascular leakage, drusen, microaneurysms and macular ischemia. Early onset drusen was reported in our previous study [8].

Previous studies have reported ocular involvement in *NLRP3*-AID, including conjunctivitis, papilledema, optic atrophy, keratitis, uveitis, cataract and glaucoma [5–7, 9]. The most common ocular involvements in *NLRP3*-AID are conjunctivitis and papilledema, which are consistent with our study findings. However, although many kinds of ocular manifestations have been reported in *NLRP3*-AID patients, retinal changes have rarely been mentioned. Previous studies have reported retinal vasculitis, retinal atrophy, chorioretinitis, cystoid macular edema and epiretinal membrane in *NLRP3*-AID patients [7, 10]. An epiretinal membrane was also found in our study. Additionally, retinal vascular sheathing was detected in previous studies,[10, 11] where it was associated with papilledema but not real retinal change.

Although the retinal findings and *NRLP3* variants differed among in these patients, there may be some commonalities. For patients with retinal findings, retinopathies were not their only signs; indeed, they all demonstrated other ocular abnormalities, such as conjunctivitis, papilledema and uveitis. This inspired us to perform fundus examinations for *NLRP3*-AID patients who already showed any ocular abnormalities. Additionally, the patients in this study were all Chinese. However, it is not clear whether retinopathy is related to race.

Notably, 3 out of 5 patients with retinal changes showed a normal retina appearance on ophthalmoscope examination, except for one patient with drusen and one patient with a focal epiretinal membrane. The visual acuity of most patients with ocular manifestations was generally good. Our study suggested that retinal changes could be detected on FA or OCT/OCTA examination in patients with good visual acuity and a normal retinal appearance on fundus photography. This indicates the importance of multimodal ophthalmic imaging, including FA,[12] OCT/OCTA imaging,[13] and ultra-widefield imaging in NLRP3-AID patients.

The NLRP3 inflammasome is a major component of innate immunity, playing a critical role in the inflammatory response. It provides a molecular platform that can be activated by multiple endogenous and exogenous stimuli, including ATP, microbial agonists, particulate matter and pore-forming toxins [14–18]. Researchers have suggested that the NLRP3 inflammasome contributes to endothelial dysfunction and causes vascular injury [19–22]. Our study found retinal vascular impairment in *NLPR3*-AID patients, such as vascular leakage and macular ischemia. Chronic inflammation could explain the vascular damage, and the presence of a focal epiretinal membrane may also be secondary to chronic inflammation. We proposed conjectures on the relationship between drusen and *NLPR3*-AID in our previous study [8].

With respect to treatment, therapeutic intervention might not be required for epiretinal membranes, drusen and microaneurysms. Patients with peripheral retinal vascular leakage and good visual acuity could be seen at regular intervals to monitor the progression of the condition. Patient 4 agreed to local and systemic treatments because his peripheral retinal vascular leakage reached 360 degrees, even though his vision was good. Scattered laser treatment at the peripheral retina vascular leakage area and sub-Tenon’s capsule injection of 20 mg triamcinolone acetonide were performed bilaterally, but there was no improvement in the vascular leakage. His clinical symptoms improved remarkably after subcutaneous administration of 150 mg of canakinumab every 8 weeks,[23] but the vascular leakage remained unaffected. Therefore, further research is needed to elucidate whether and how peripheral retina vascular leakage can be treated.

Our study is limited by the small sample size due to the rarity of *NLRP3*-AID. Additionally, the follow-up periods were not long enough to observe the progression of the retinal changes. Despite the above limitations, this is the largest cohort study of *NLRP3*-AID in China, and the first study to report four new retinal changes in *NLRP3*-AID.

## Conclusions

Peripheral retinal vascular leakage, macular ischemia, microaneurysms and drusen are newly identified retinal findings in patients with *NLRP3*-AID, which increased the understanding of this rare disease. It also suggests the importance of detailed retinal examination in *NLRP3*-AID patients.

## Data Availability

The datasets used and/or analysed during the current study are available from the corresponding author on reasonable request.
